# The impact of gut microbiome enterotypes on ulcerative colitis: identifying key bacterial species and revealing species co-occurrence networks using machine learning

**DOI:** 10.1080/19490976.2023.2292254

**Published:** 2023-12-20

**Authors:** Xuangao Wu, Ting Zhang, TianShun Zhang, Sunmin Park

**Affiliations:** a Department of Bioconvergence, Hoseo University, Asan, Korea; b Department of Food and Nutrition, Obesity/Diabetes Research Center, Hoseo University, Asan, Korea

**Keywords:** Ulcerative colitis, enterotypes, gut microbiome, *R. gnavus*, *O. splanchnicus*, *B. uniformis*, machine learning, co-occurrence network

## Abstract

Ulcerative colitis (UC) is a chronic inflammatory intestinal disease affecting the colon and rectum, with its pathogenesis attributed to genetic background, environmental factors, and gut microbes. This study aimed to investigate the role of enterotypes in UC by conducting a hierarchical analysis, determining differential bacteria using machine learning, and performing Species Co-occurrence Network (SCN) analysis. Fecal bacterial data were collected from UC patients, and a 16S rRNA metagenomic analysis was performed using the QIIME2 bioinformatics pipeline. Enterotype clustering was conducted at the family level, and deep neural network (DNN) classification models were trained for UC and healthy controls (HC) in each enterotype. Results from eleven 16S rRNA gut microbiome datasets revealed three enterotypes: Bacteroidaceae (ET-B), Lachnospiraceae (ET-L), and Clostridiaceae (ET-C). *Ruminococcus (R. gnavus)* abundance was significantly higher in UC subjects with ET-B and ET-C than in those with ET-L. *R. gnavus* also showed a positive correlation with *Clostridia* in UC SCN for ET-B and ET-C subjects, with a higher correlation in ET-C subjects. Conversely, *Odoribacter* (*O.) splanchnicus* and *Bacteroides (B.) uniformis* exhibited a positive correlation with tryptophan metabolism and AMP-activated protein kinase (AMPK) signaling pathways, while *R. gnavus* showed a negative correlation. In vitro co-culture experiments with *Clostridium (C.) difficile* demonstrated that fecal microbiota from ET-B subjects had a higher abundance of *C. difficile* than ET-L subjects. In conclusion, the ET-B enterotype predisposes individuals to UC, with *R. gnavus* as a potential risk factor and *O. splanchnicus* and *B. uniformis* as protective bacteria, and those with UC may have ultimately become ET-C.

## Introduction

Ulcerative colitis (UC) is a chronic inflammatory disease affecting the intestinal tissue and is characterized by remission and relapse.^1^ It can progress from a mild asymptomatic stage to widespread colon inflammation, leading to bloody stools, colonic motility dysfunction, tissue damage, and fibrosis.^1^ UC is also viewed as an overly aggressive mucosal immune response to specific gut dysbiosis, a condition marked by abnormalities in the gut microbiota composition, a disruption of the mucus surface barrier homeostasis, and an increase in bacterial infections of the previously sterile mucus layer.^2^ The total incidence and prevalence of UC vary between regions, with the highest incidence in European countries (1.6–11.9 per 10,000 people) and South Korea (about 3.62 per 10,000 people).^3,4^

The gut microbiome is crucial in shaping the colonic environment, regulating host signaling, immunomodulation, and producing antimicrobial substances.^5^ It influences the host’s metabolic pool and produces various metabolites, including short-chain fatty acids (SCFAs).^5^ The SCFAs activate the G protein-coupled receptors on macrophages, dendritic cells, and mast cells, thereby regulating the release of anti-inflammatory and pro-inflammatory cytokines.^6^ Butyric acid serves as the primary energy source for intestinal epithelial cells, promotes regulatory T cell development, and enhances mucus secretion by the goblet cells to maintain the intestinal mucosal barrier.^7^ Damage due to a depleted mucus barrier is believed to contribute to the development of UC.^2^ Gut microbes also regulate human amino acid metabolism.^5^ Tryptophan metabolites produced by the gut microbiome can enhance the intestinal barrier function by promoting zonula occludens-1 (ZO-1) expression.^8^

In 2011, Arumugam et al. introduced the concept of enterotypes, enabling gut microbe clustering and grouping for research purposes.^9^ Enterotypes can be broadly categorized into Bacteroides, Prevotella, and Ruminococcus.^9^ However, studies using different clustering and grouping strategies have produced heterogeneous results and specific enterotypes have been linked to diseases such as rectal cancer and diabetes, among others.^10,11^

The rapid development of high-throughput sequencing has led to a significant increase in gut microbiome data related to diseases. The machine learning approach has become an effective method to identify disease-related gut microbiome markers through data mining, with applications in cancer, diabetes, and dementia research.^12^ The gut microbiome is a complex ecosystem involving interactions like cross-feeding, competition, and symbiosis.^13^ Despite this, most gut microbiome research focuses on classification and functional responses, with little understanding of the influence of gut microbiome interactions on diseases or the interactions between enterotypes in different diseases.

In this study, we innovatively used deep neural network (DNN) classification models to analyze the UC data from public databases, delving deeper into the differences in symbiotic bacteria affecting UC among various enterotypes. We identified gut microbiome markers and their symbiotic relationships with other gut microbiomes in UC subjects and healthy controls (HC) of different enterotypes.

## Results

### Study selection

We included 11 studies on the gut microbiome of UC subjects and HC, with study populations comprising American, Japanese, and Chinese cohorts (Table 1). Each study had a small sample size. All data were downloaded for pooled 16S rRNA metagenomic analysis. The fecal bacteria data for 10 UC studies with and without HC were selected for a combined study. However, two studies (PRJNA541040 and PRJNA50637) did not contain HC, and NIH HMP included large numbers of UC patients from the USA. The considerable disparity in the ratio of UC to HC groups in the collected data arose from the fact that the PRJNA50637 data included a large amount of data from American UC patients but not from HC. The HC was added from the studies (PRJNA296920 and PRJNA386260) for inflammatory bowel disease, and the participants in both projects corresponded to PRJNA386260, which was conducted in the USA and were matched for age.

**Table 1.** Information on the studies included in the meta-analysis.

Study	Accession number	Design	Sample grouping	Age	Gender	Country	Sampling	DNA extraction	16S region	Seq Tech
Alam et al.	PRJEB33711	Case–control	UC: 11 Health: 10	NA	NA	UK	Collect feces in sterile tubes and immediately freeze at −80°C	FastDNA Spin Kit for Soil	V1–3	454 Sequencing
NIH HMP	PRJNA50637	Cross-sectional	UC: 601	NA	NA	USA	NA	NA	V1–3	454 Sequencing
NA	PRJDB6133	Case–control	UC:30 Health:23	NA	NA	JAPAN	NA	MiSeq 600 cycle v3 kit	V3–4	MiSeq
NA	PRJNA368966	Case–control	UC:26 Health:13	NA	NA	CHINA	NA	NA	V5-V6	MiSeq
NA	PRJNA296920	Case–control	Health: 362	NA	NA	USA	NA	NA	V5-V6	MiSeq
Weng et al.	PRJNA431126	Case–control	UC:107 Health:42	43.4±0.94	Male:97 Feaml:52	CHINA	Collect fecal samples with the help of a stool sampling kit and stored at home at 4 to 8°C for up to 24 hours	DNA Extraction Kits	V4	MiSeq
Maldonado‐Arriaga et al.	PRJNA596546	Case–control	UC:18 Health:15	37.1±1.36	Male:19 Female:14	Mexico	Collect the first-morning stool in a sterile stool collection tube and store the collected sample at −80°C	AllPrep PowerFecal DNA	V3-V6	MiSeq
Dai et al.	PRJNA681685	Case–control	UC:10 Health:14	30±0.88	Male:12 Femal:12	CHINA	Stool samples were collected in the Gastroenterology Department of Xijing Hospital and stored at −80°C	E.Z.N.A. stool DNA Kit	V3-V4	MiSeq
Wan et al.	PRJNA753210	Case–control	UC:29 Health:30	39 people>age 45	Male:39 Female:20	CHINA	Stool samples were collected in the Gastroenterology Department of Xijing Hospital and stored at −80°C	E.Z.N.A. stool DNA Kit	V3-V4	MiSeq
Cao et al.	PRJNA541040	Cross-sectional	UC:11	15–65	Male:7 Female:4	CHINA	NA	NA	NA	HiSeq 2500
Lloyd-Price J et al.	PRJNA398089	Case–control	UC:30 Health:26	28.8±4.04	Male:10 Female:45	USA	NA	NA	NA	HiSeq 2000
NA	PRJNA386260	Case–control	Health:211	55.3±1.39	Male:73 Female:138	USA	NA	NA	V4	Illumina

The table describes the design format in each study, the nationality of the study subjects, the amplified segment of 16s rRNA, and the system used for sequencing. Parts not provided are denoted by NA. Ulcerative colitis, UC; healthy control, HC.

A total of 1619 samples were analyzed, including 873 from the UC cohort and 746 from the HC. QIIME2‘s DADA2 was uniformly employed for quality control to avoid the impact of different plugins and programs on the quality control of various sequences. After the QIIME metagenomic analysis of 16S rRNA, 1328 samples remained, comprising 621 from the UC cohort and 707 from the HC (Table 1). Considering that our study included eight different data sets, we plotted principal coordinates analysis (PCoA) to observe potential cohort-driven bias/effects in the results and statistically identified potential batch effects (*P* < 0.001). According to the distribution of the PCoA diagram, it was found that the batch effects were mostly limited to the PRJNA50637 study of UC alone and the PRJNA296920 and PRJNA386260 studies of HC alone, while the data of other studies were in between three studies. Depending on the method, batch effect correction tools for microbiome data usually require metadata from each study, including case-control groups. However, since the relevant information was limited in the public data,^14–16^ batch correction was unable to be performed. Gut communities had robust properties, and slight perturbations usually had little effect on the overall structure of microbial networks, and it might have been a reasonable strategy to integrate networks and observe small changes in network submodules (Supplementary Figure S1).^15^

### Enterotype and biodiversity analysis

**Figure 1.**
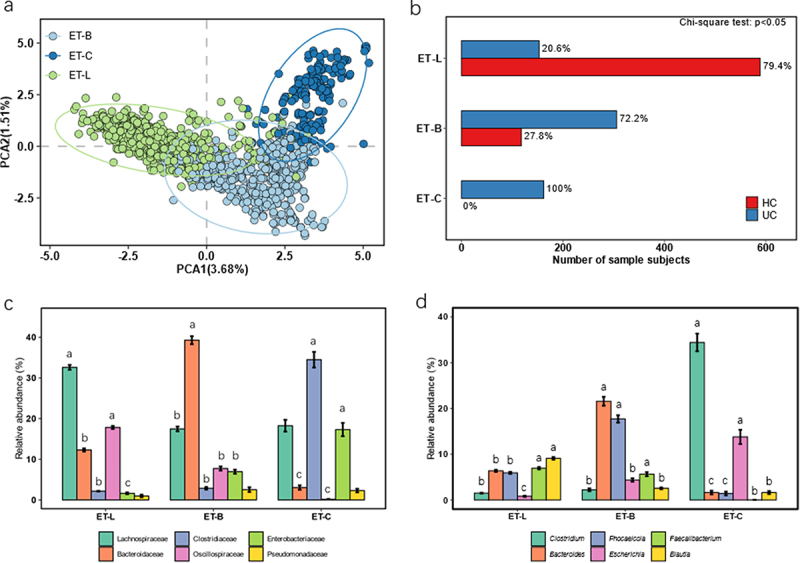
Characterization and distribution of three enterotypes in UC and HC subjects based on the gut microbiome. Ulcerative colitis, UC; healthy control, HC; ET-B, enterotype Bacteridaceae; enterotype ET-L, Lachnospiraceae; ET-C, enterotype Clostridiaceae. (a) The principal component analysis (PCA) diagram of the three enterotypes was drawn based on the fecal microbes at the family level. (b) The number of UC and HC subjects in each enterotype. The Chi-square test was used to count the significant differences in the number of people among the various enterotypes. (c) The relative abundance of the top 6 family taxon in each enterotype, we employed Tukey’s post-hoc test to identify significant differences among the enterotypes. Taxa that exhibited significant differences were annotated with English letters for clarity. (d) The relative abundance of the top 6 genus taxon in each enterotype, we employed Tukey’s post-hoc test to identify significant differences among the enterotypes. Taxa that exhibited significant differences were annotated with English letters for clarity.

In enterotype analysis, the optimal number of clusters was found to be three. The genus-level principal component analysis (PCA) plots of enterotypes^9^ from all cohorts showed that each enterotype was well separated when divided into three groups (Figure 1a). We compared the dominant taxa of the three enterotypes at both the family and genus levels. At the family level, we identified significantly dominant taxa for the other three enterotypes as Lachnospiraceae, Bacteroidetes, and Clostridiaceae, respectively (Figure 1c, *P* < 0.05). At the genus level, *Bacteroides* and *Clostridium* were identified as significantly dominant genera for the two enterotypes (Figure 1d, *P* < 0.05). The three enterotypes identified were named after the primary bacteria at the family level, such as enterotype Bacteridaceae (ET-B), Lachnospiraceae (ET-L), and Clostridiaceae (ET-C) (Figure 1c). After applying the Chi-square test for statistical analysis, we observed significant differences in the distribution of enterotypes between the UC and HC groups. We calculated the proportion of the UC group in ET-C, ET-B, and ET-L. The proportions of UC among the ET-C, ET-B, and ET-L enterotype groups were 100%, 72.2%, and 20.5%, respectively (Figure 1b). Based on the Chi-Square tests test, the proportion of UC patients was significantly different among the ET-B, ET-L, and ET-C enterotypes (Figure 1b, *P* < 0.05).

**Figure 2.**
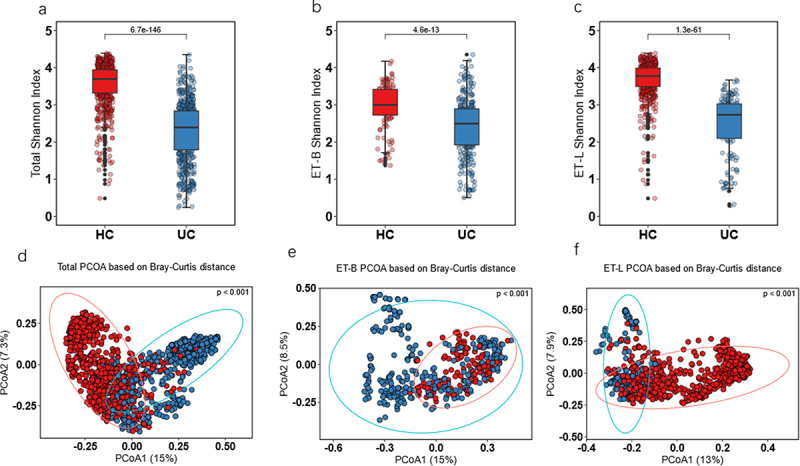
α-diversity by Shannon index and β-diversity by Bray–Curtis method. (a) α-diversity Shannon index in total participants. (b) α-diversity Shannon index in ET-B. (c) α-diversity Shannon index in ET-L. (d) β-diversity in total participants. (e) β-diversity in ET-B. (f) β-diversity in ET-L. Shannon index used the Wilcoxon test for significance analysis, and β-diversity used permutation multivariate analysis of variance (PERMANOVA) for significance. The numbers presented a significance level. Ulcerative colitis, UC; healthy control, HC; ET-B: Enterotype Bacteroidaceae cohort, ET-L: Enterotype Lachnospiraceae cohort; PCoA: Principal coordinate analysis.

The Shannon diversity indices in the HC subject group were significantly higher than those in the UC group in total participants (Figure 2a, *P* < 0.05). We also observed significantly higher Shannon diversity indices in HC than UC subjects in the enterotype subgroups with ET-B and ET-L (Figure 2b,c, *P* < 0.05). In the PCoA plot based on the Bray-Curtis distance, the UC and HC groups were well-separated, with permutational multivariate analysis of variance (PERMANOVA) statistics indicating significant differences between the groups in total participants (Figure 2d, *P* < 0.05). In the β-diversity results of ET-B, we observed a certain degree of overlap between HC and UC, distinctly different from the clear separation in ET-L (Figure 2e, *P* < 0.05). However, even though HC samples overlap with UC samples in some respects, their microbial community diversity was significantly higher than that of UC samples. The HC participants who overlapped with the UC cluster might be susceptible to becoming UC, and the overlap between HC and UC was higher in ET-B, which might have a greater risk of developing UC. In the ET-L enterotypes, the UC and HC groups were also well-separated in the PCoA plot based on the Bray-Curtis distance. PERMANOVA statistics demonstrated significant differences (Figure 2f, *P* < 0.05). These observations suggested that the alterations in the disease state could vary between different enterotypes. Such differences may not be fully captured by β-diversity alone. Instead, further network analysis might require observing the community relationships among different enterotypes.

### DNN classification model composition and performance

After attempting various settings, the classification model trained with the above configurations achieved the best performance. The receiver operating characteristic curve (AUC) values of the DNN models for the total, ET-B, and ET-L participants ranged from 0.93 to 0.96. The accuracy values varied from 0.88 to 0.93, sensitivity values ranged from 0.78 to 0.94, specificity values varied from 0.71 to 0.97, precision values ranged from 0.86 to 0.89, and F1 values varied from 0.81 to 0.91 (Supplementary Figure S2 and Table S1).

### SHapley additive exPlanations(SHAP) interpreter and network analysis in the total cohort

We obtained the top 20 species of microorganisms considered important in the DNN classifier through the SHAP interpreter and determined their classification bias toward UC or HC (Supplementary Figure S3a). A total of 15 bacteria were skewed toward the HC group, and 5 bacteria toward the UC group (Supplementary Figure S3a). In the SHAP interpreter of the ET-B cohort, the top two critical UC-associated species were *Fusicatenibacter saccharivorans (F. saccharivorans)* and *Ruminococcus gnavus (R. gnavus)*, whereas the HC-associated species were *Oscillibacter valericigenes (O. valericigenes)* and *Bacteroides faecis (B. faecis)*. Box plots were generated for 20 taxons, and the Wilcoxon rank test was conducted (Supplementary Figure S3b). Significant differences were observed for all taxons (*P* < 0.05; Supplementary Figure S3b). We visualized the bacteria selected by the DNN in the species co-occurrence network (SCN), which revealed that in the HC group, the bacterial network was formed of tightly connected symbiotic bacteria. *Faecalibacterium prausnitzii (F. prausnitzii)*, a common butyric acid-producing bacterium, had 17 connections (Supplementary Table S2). We also added the absolute correlation coefficient value for each bacterium to evaluate its role in the network. The maximum sum of the correlation coefficient values for *F. prausnitzii* was 6.85, indicating its potential importance in the co-occurrence network of the HC group (Supplementary Table S2). The symbiotic bacteria, *including Erysipelatoclostridium ramosum (E. ramosum)* and *R. gnavus*, in the UC group displayed dense negative connections, while *Loriellopsis cavernicola (L. cavernicola*) displayed negative connections in the HC group (Supplementary Figure S3c). Among them, *E. ramosum* and *R. gnavus* had the highest number of connections and were closely positively correlated with each other (Supplementary Figure S3c).

### SHAP interpreter and network analysis in the ET-L cohort

**Figure 3.**
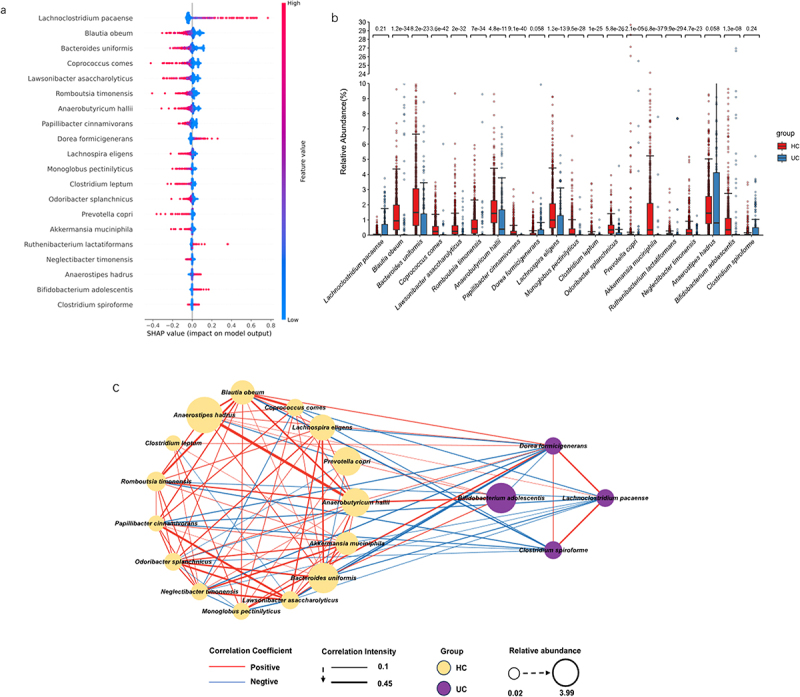
The SHapley additive exPlanations (SHAP) interpreter and species co-occurrence network (SCN) of the enterotype Lachnospiraceae (ET-L) queue deep neural network (DNN). (a) The SHAP interpreter was used to conduct the microbial-specific importance analysis in the DNN classifier. The middle line is biased to the left for the healthy controls (HC) classification and vice versa for the ulcerative colitis (UC) classification. The color of the scatter points represents the influence of the relative abundance of the feature on the classification. The variables used to train the DNN network were species of microorganisms significantly different in the UC and HC groups. (b) Box plot of the 20 taxons included in the SHAP beeswarm plot and the numbers indicated the significance level in the corresponding Wilcoxon rank sum test. (c) The SCN was constructed using important gut microbes in the DNN classifier. The SHAP importance was used to determine the top 20 bacteria, and the network diagram was drawn. It was determined whether it belonged to UC or HC according to the SHAP bee colony diagram and the mean value of UC and HC. All connections with a sparse correlation for compositional data (SparCC) correlation coefficient less than 0.1 were removed in the SCN. The red edges represent positive correlation, the blue represents negative correlation, the thickness represents the size of the absolute correlation coefficient, the yellow node represents the HC group, the purple represents the UC group, and the node size represents the relative abundance.

We also trained the DNN classifier in the ET-L cohort alone and obtained the top 20 primary species of bacteria. The bacteria in the ET-L cohort of the UC group identified by DNN were entirely different from those in the total group (Figure 3a). Box plots were generated for 20 taxons, and significant differences were observed for all taxons except *Lachnoclostridium pacaense (L. pacaense)* and *Clostridium spiroforme* (*C. spiroforme*) (*P* < 0.05 with Wilcoxon rank test; Figure 3b). The results suggest that a distinct ecological network of pathogenic bacteria may influence their growth and survival in different enterotypes (Figure 3c). Among them, in the UC group, *Dorea formicigenerans (D. formicigenerans)* had the maximum number of connections, with up to 14 connections in the SCN (Supplementary Table S3). In the HC group, *Odoribacter splanchnicus (O. splanchnicus)* and *Bacteroides uniformis* (*B. uniformis)* had the highest number of connections, with 13 connections each (Supplementary Table S3). The results suggested that *O. splanchnicus* and *B. uniformis* might be protective against UC.

### SHAP interpreter, network analysis, and communities by reconstruction of unobserved states (PICRUSt2) function prediction results in the ET-B cohort

**Figure 4.**
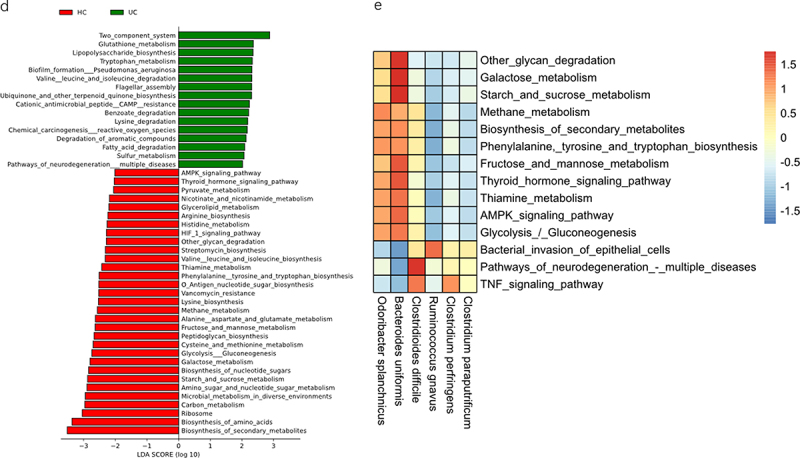
(Continued).

In ET-B’s SHAP interpreter, the top two abundant bacteria in UC were *Desulfovibrio simplex* (*D. simplex)* and *R. gnavus*, while those in HC were *O. splanchnicus* and *B. uniformis*. Notably, *R. gnavus* also appeared in the SHAP interpreter of the total participants (Figure 4a). Box plots were generated for 20 taxons, and significant differences were observed for all taxons (*P* < 0.05 in the Wilcoxon test; Figure 4b). As seen in the results presented in section 3.2, the proportion of UC in the ET-B cohort was higher than that in the ET-L cohort, and there were no HCs in the ET-C cohort. Therefore, we conducted a network analysis between ET-B and the common pathogenic bacterium, *Clostridium spp*. (Figure 4c). *R. gnavus* was positively correlated with *Clostridium difficile (C. difficile)*, *C. paraputrificum*, and *C. perfringens* (Figure 4c). *O. splanchnicus* and *B. uniformis* were also identified as important bacteria in HC in the ET-L cohort (Figure 4b). In the HC group, *B. uniformis* had the highest number of connections and the highest correlation coefficient (3.94) in the SCN (Supplementary Table S3). In the SCN, *B. uniformis* was positively correlated with *O. splanchnicus* and negatively correlated with *R. gnavus*, *C. difficile*, *C. paraputrificum*, and *C. perfringens* (Figure 4c).
**Figure 4.**
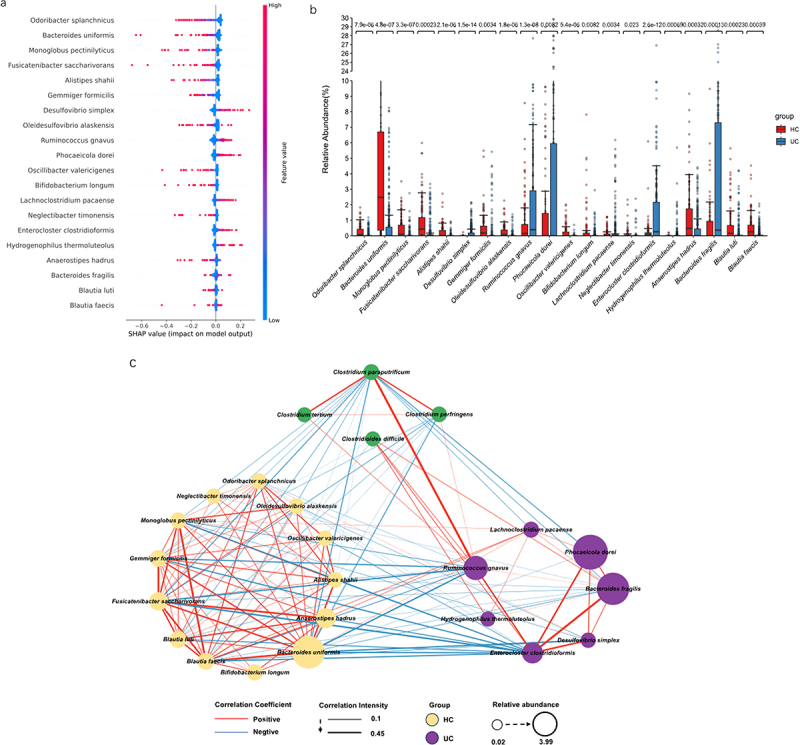
The SHapley additive exPlanations (SHAP) interpreter and species co-occurrence network (SCN) of the enterotype Bacteroides (ET-B) queue deep neural network (DNN). (a) The SHAP interpreter was used to conduct the microbial-specific importance analysis in the DNN classifier. The middle line is biased to the left for the healthy controls classification (HC) and vice versa for the ulcerative colitis (UC) classification. The color of the scatter points represents the influence of the relative abundance of the feature on the classification. The variables used to train the DNN network were species of microorganisms significantly different in the UC and HC groups. (b) Box plot of the 20 taxons included in the SHAP swarm plot and the numbers indicated the significance level in the corresponding Wilcoxon rank sum test. (c) The SCN was constructed using important gut microbes in the DNN classifier. The SHAP importance was used to determine the top 20 bacteria, and the network diagram was drawn. It was determined whether it belonged to UC or HC according to the SHAP bee colony diagram and the mean value of UC and HC. All connections with a sparse correlation for compositional data (SparCC) correlation coefficient less than 0.1 were removed in the SCN. The red edges represent positive correlation, the blue represents negative correlation, the thickness represents the size of the absolute correlation coefficient, the yellow node represents the HC group, the purple represents the UC group, and the node size represents the relative abundance. (d) Linear discriminant analysis (LDA) scores of the HC and UC groups in LDA effect size (LEfSe). (e) Differential analysis of PICRUSt2-predicted gut microbiome function between UC and HC in the ET-B cohort was performed using LEfSe. The correlation analysis between the functions that LEfSe showed significant differences and the common pro-inflammatory functions and the important bacteria of HC and UC in the SHAP analysis was drawn into a heatmap.

Gut microbes corresponding to the HC and UC groups were selected by Linear Discriminant Analysis (LDA) Effect size (LEfSe) analysis (Figure 4d). The metabolic function of gut microbes selected for each group, as assessed by PICRUSt2, revealed significant differences in microbial biosynthesis, metabolism, and signal transduction between the UC and HC groups (Figure 4e). While drawing a correlation heatmap, *B. uniformis* and *O. splanchnicus* were highly positively correlated with phenylalanine, tyrosine, and tryptophan biosynthesis, the AMPK signaling pathway, fructose and mannose metabolism, and thiamine metabolism. However, the *R. gnavus*, *C. perfringens*, *C. paraputrificum*, and *C. difficile* in the UC were positively correlated with the neurodegeneration pathways – bacterial invasion of epithelial cells, and the tumor necrosis factor (TNF) signaling pathway (Figure 4e).

### ET-C enterotype network analysis

Among ET-L, ET-B, and ET-C, the ET-C included only UC patients, suggesting that an increase in the relative abundance of *Clostridiaceae* in gut microbes may be a primary risk factor for UC. ET-C is not a typical enterotype for healthy people and could occur in certain disease conditions. The ET-B had a higher proportion of UC patients than ET-L, similar to the results of a previous study^17^ (Figure 1b). Therefore, we focused on the bacteria in the ET-C group for comparison with those in the ET-B using SCN. We first compared the relative abundance of important UC and HC microbes of ET-B in each enterotype (Table 2). *B. fragilis*, *Phocaeicola dorei*, and *D. simplex* were significantly higher in ET-B than in the other two enterotypes in the UC group (Table 2, *P* < 0.05). *R. gnavus* was significantly higher in ET-B and ET-C than in ET-L, with the relative abundance in ET-C being 0.52 higher than in ET-B (Table 2, *P* < 0.05). *Anaerostipes hadrus (A. hadrus)*, *Bifidobacterium longum (B. longum)*, *Bacillus luti (B. luti)*, *F. saccharivorans*, and *O. splanchnicus* in HC were significantly higher in ET-L than in the other two enterotypes, with *O. splanchnicus* in ET-B being significantly higher than in ET-C (Table 2, *P* < 0.05). *B. uniformis* in ET-B and ET-L were significantly higher than in ET-C (Table 2, *P* < 0.05).

**Table 2.** The relative abundance of ET-B group UC bacteria and HC bacteria in each enterotype.

	Gut microbes	ET-L(*n*=742)	ET-B(*n*=424)	ET-C(*n*=162)
UC	*Bacteroides fragilis*	0.49±0.23^b^	5.12±0.51^a^	0.55±0.26^b^
	*Desulfovibrio simplex*	0.16±0.03^a^	0.19±0.02^a^	0.01±0^b^
	*Enterocloster clostridioformis*	0.08±0.03^b^	2.23±0.25^a^	0.19±0.06^b^
	*Lachnoclostridium pacaense*	0.55±0.08^a^	0.33±0.05^b^	0.12±0.06^c^
	*Phocaeicola dorei*	0.53±0.14^b^	5.25±0.55^a^	0.13±0.05^b^
	*Ruminococcus gnavus*	0.57±0.13^b^	2.95±0.31^a^	3.47±0.44^a^
HC	*Anaerostipes hadrus*	2.77±0.33^a^	0.51±0.06^b^	0.08±0.04^b^
	*Bifidobacterium longum*	1.01±0.28^a^	0.07±0.02^b^	0.79±0.26^a^
	*Blautia luti*	1.07±0.19^a^	0.19±0.03^b^	0±0b
	*Fusicatenibacter saccharivorans*	2.26±0.28^a^	0.23±0.04^b^	0.13±0.07^b^
	*Odoribacter splanchnicus*	0.17±0.04^a^	0.07±0.01^b^	0±0c
	*Bacteroides uniformis*	2.4±0.9^a^	6.17±0.87^a^	0.007±0.03^b^
Clostridium	*Clostridioides difficile*	0±0	0.03±0.0^3^	0.07±0.03
	*Clostridium butyricum*	0.03±0.02^b^	0.78±0.25^b^	8.52±1.35^a^
	*Clostridium paraputrificum*	0.04±0.04^b^	0.4±0.07^b^	1.65±0.3^a^
	*Clostridium perfringens*	0.29±0.12^b^	0.29±0.06^b^	6.48±0.89^a^
	*Clostridium saudiense*	0.03±0.02^b^	0.24±0.08^b^	3.4±0.69^a^
	*Clostridium tertium*	0.12±0.08^b^	0.07±0.04^b^	0.7±0.16^a^

^a, b, c^Different superscript letters in each gut microbe indicated significant difference among the groups by Tukey post hoc test at *P* < 0.05.

The relative abundance of ulcerative colitis (UC) microbes and healthy control (HC) microbes obtained in the ET-B cohort in each enterotype disease group was compared.

When the bacterial networks in UC were compared, it was found that the HC bacteria, *Akkermansia muciniphila (A. muciniphila)*, *B. faecis*, *B. uniformis*, *Parabacteroides merdae (P. merdae)*, *Gemmiger formicilis (G. formicilis)*, *Lachnospira eligens (L. eligens)* were depleted in the ET-C UC network, compared to the ET-B UC network (Table 2 and Supplementary Figure S4). The abundance of the remaining microorganisms in the HC group was also significantly reduced in ET-C (Table 2 and Supplementary Figure S4). *R. gnavus* was identified as a harmful microorganism in UC in the previous ET-B analysis, and the same trend was also observed in the ET-C analysis. In the ET-B analysis, *R. gnavus* was positively correlated with all *Clostridium* species, and this positive correlation was enhanced in ET-C. *R. gnavus* was the only microorganism whose abundance did not decrease in ET-C among the UC dominant bacteria, thus showing a symbiotic relationship with *Clostridium* (Table 2 and Supplementary Figure S4). Therefore, *R. gnavus* may contribute to increased *Clostridium* bacteria in UC patients with ET-B and exacerbate gut microbiome dysbiosis.

### In vitro co-culture experiments with C. difficile and different enterotypes

**Figure 5.**
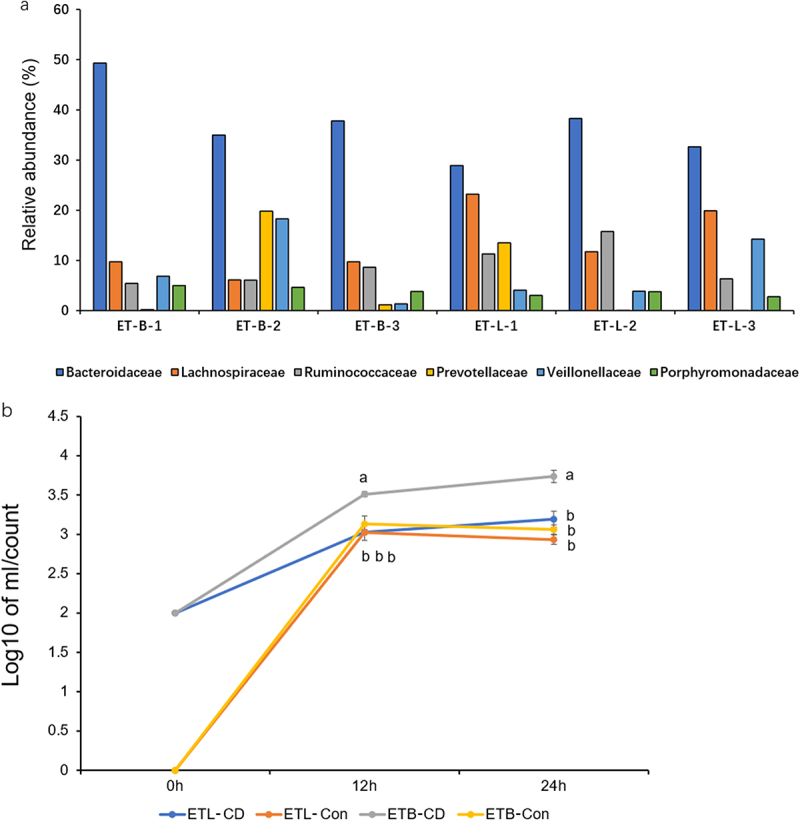
Gut microbiota composition of volunteer subjects and results of *in vitro* experiments. (a) The relative abundance of the top 5 intestinal flora at the family level of 5 subjects. Subjects with a higher abundance of *Bacteroidaceae* and lower numbers of Lachnospiraceae and *ruminococcaceae* were classified as enterotype bacteridaceae (ET-B). Subjects with a higher abundance of *Lachnospiraceae* and *ruminococcaceae* were classified as enterotype Lachnospiraceae (ET-L). (b) Enumeration of *C. difficile* in ET-B and ET-L feces cultured *in vitro*. ETL-CD: ET-L enterotype feces co-cultured with *C. difficile*, ETL-Con: ET-L enterotype feces co-cultured with *C. difficile*, ETB-CD: ET-B enterotype feces co-cultured with *C. difficile*, ETB-Con: ET-B enterotype feces cultured alone. Statistical differences in Tukey’s test are expressed in letters. Different letters indicate significant differences between groups, and the same letters indicate no significant differences.

Finally, we recruited 6 males and 4 females, a total of 10 healthy volunteers aged 25.2 ± 0.66, and they had not taken antibiotics and probiotics for over 2 weeks. After analyzing the gut microbiome using 16S rRNA amplicon sequencing of fecal samples from the 10 subjects, we identified 6 participants having ET-B and ET-L for our study. Three participants were categorized as ET-B, and the other three as ET-L (Figure 5a). The C. difficile contents using qPCR were plotted using a regression curve to quantify the *C. difficile* count after co-culture of fecal bacteria and gradient-diluted *C. difficile*. Using qPCR, we were able to detect *C. difficile* from a range of 1.48 × 10^2^ to 1.48 × 10^8^, with an R^2^ value of 0.993. The *in vitro* co-culture experiment results showed that at 12 and 24 hours, the fecal bacteria from ET-B had a significantly higher abundance of *C. difficile* than those from ET-L (Figure 5b, *P* < 0.05).

## Discussion

Enterotypes can be potentially used to stratify human cohorts in clinical study designs based on gut microbiota composition, with each enterotype having a different bacterial composition. Enterotypes can be useful in studying biologically relevant community characteristics.^18^ In research on IBD, among the four enterotypes clustered by the Dirichlet multinomial mixtures (DMM) method, it was found that the prevalence of IBD in the Bacteroides enterotype was as high as 80%.^17^ In the present study, the results showed a high prevalence of UC in ET-B. *R. gnavus* is a common commensal bacterium found in the digestive tracts of more than 90% of humans. However, studies have shown that the relative abundance of *R. gnavus* increases briefly and rapidly in IBD, reaching a peak of 69% maximum relative abundance.^19^ *R. gnavus* is reported to produce a potent inflammatory polysaccharide with a rhamnose backbone and short glucose side chains, a glucan that increases TNF-α and toll-like receptor 4 (TLR4) immune responses to produce inflammation.^20^ A distinct feature of gut microbiota dysbiosis in IBD is a shift in the gut microbes toward organisms that can cope with increased oxidative stress.^21^ Butyrate produced by microbes can stimulate the intestinal epithelial cell line to consume more oxygen and stabilize the expression of hypoxia-inducible factors (HIFs).^22^ HIFs are essential transcription factors that protect the intestinal barrier and promote higher oxygen consumption by the colon cells through the β-oxidation metabolic pathway.^22^ Although *R. gnavus* is classified as an obligate anaerobic bacterium, it has a certain oxygen tolerance.^19^ After 1 hour and 3 hours of oxygen exposure, *R. gnavus* retains a 10^6^ and 10^4^ bacterial count, respectively.^19^ This may also explain the increased proportion of *R. gnavus* in UC.

*R. gnavus*, as an intestinal commensal bacterium, exhibited the ability to decompose mucin.^23^ The ability of *R. gnavus* to decompose mucin is strain-specific. *R. gnavus* ATCC 29,149 exhibits a unique sialic acid metabolism pathway to produce 2,7-anhydro-neu5Ac derivatives.^24^ Sialic acid (N-acetylneuraminic acid (Neu5Ac)) is commonly found in the terminal location of colonic mucin glycans and thus serves as a nutrient for *R. gnavus*. The gut microbiome has evolved to adhere to mucus barriers, including adhesins, flagella, and pili, and cross-feed through mucin degradation.^2^ The monosaccharides produced by the chemical degradation of mucin attract the colonization of harmful microorganisms such as *C. difficile*. ^25^ In the SCN analysis of the present study, *R. gnavus* was positively correlated with *C. difficile*, indicating that it may form a cross-feeding relationship with *C. difficile* by degrading mucin to promote the colonization of *C. difficile*.

As multi-omic studies of IBD continue to advance, our understanding of how gut microbial metabolites affect the host gut and how these metabolites affect disease progression and etiology in IBD become clearly increasing.^5,26,27^ In IBD metabolomics studies, a general trend toward decreased production of SCFAs is found. The decreased butyrate production is also consistent with a decreased presence of butyrate producers *F. prausnitzii* and *R. hominis*.^27^ In the present study, butyrate producers such as *A. hadrus* and *O. splanchnicus* were significantly reduced in the ET-B UC group.^28,29^ Intestinal inflammation in IBD patients is an adaptation of the intestinal environment to oxidative stress, and the resulting oxidative stress and changes in bile acid metabolism affect the relative abundance of butyrate-producing bacteria such as *F. prausnitzii*.^5,26^ In this study, we found that the AMPK signaling pathway was suppressed in ET-B UC intestinal microbes and that the AMPK signaling was generally negatively associated with bacteria enriched in UC and positively associated with *B. uniformis* and *O. splanchnicus*. The activation of AMPK can inhibit oxidative stress in different lesions.^30^ This might represent a gut adaptation to oxidative stress experienced by UC patients due to inflammation, resulting in a reduction in metabolically beneficial bacteria.

The results of assessing the resistance to harmful bacteria in ET-L and ET-B in *in vitro* co-culture experiments revealed that the ET-L group had greater resistance to *C. difficile* than ET-B, possibly due to a more robust beneficial symbiotic microbial ecology. The Lachnospiraceae group accounts for approximately 10% of the total gut microbiota. Some members of this group, such as *R. hominis*, have been identified as commercial probiotics and have been patented.^31^ From the perspective of metabolites and immune regulation, the Lachnospiraceae family of bacteria produces SCFAs, which play a crucial role in regulating the carbon source of intestinal epithelial cells and inducing regulatory T cells.^32^ Regarding inhibiting pathogenic bacteria, previous studies based on germ-free mice have shown that isolated strains of Lachnospiraceae can effectively suppress *C. difficile* infections.^33^

Despite the standardized bioinformatics procedures employed, the data were sourced from various studies that might have dissimilar experimental conditions, including DNA extraction methods, sequencing counts, and diverse 16s rRNA sequencing regions. This variation led to the emergence of a batch effect among the studies. However, it is noteworthy that this batch effect was more pronounced between the UC and HC groups rather than within each project, as evidenced by the PCoA plot. It suggests a degree of homogeneity within projects in terms of project-driven discrepancies. While certain potential confounding variables that could influence ulcerative colitis were not accounted for and adjusted in the analysis, the study findings were categorized based on enterotypes influenced by dietary patterns. This categorization allowed for the potential adjustment of some confounding factors. However, the nature of the study being based on case-control designs precludes the establishment of causal relationships between the identified results. Therefore, the limitations were considered when interpreting the findings and their implications.

In conclusion, this study innovatively compared gut microbial samples from UC and HC subjects using enterotype analysis, machine learning-based critical microbial screening, and SCN analysis. The findings revealed significant differences in the ratios of UC and HC across different enterotypes, with ET-B being a UC-susceptible enterotype and ET-L acting as a UC-protective enterotype. Additionally, the study identified the potential pathogen *R. gnavus*, along with potential beneficial symbionts *O. splanchnicus* and *B. uniformis* in UC. To further elucidate the actual interactions and mechanisms of these key species, future research employing *in vitro* co-culture experiments, cell-based assays, and *in vivo* studies is of critical importance.

## Methods

### Data acquisition and preprocessing

The gut microbiome metagenomic data were obtained through three approaches: 1) The data repository for Gut Microbiota (GMrepo, [https://gmrepo.humangut.info/home]);^34^ 2) The European molecular biology laboratory (EMBL)‘s European Bioinformatics Institute (EMBL-EBI, https://www.ebi.ac.uk/ena/browser/home);^35^ and 3) Google Scholar. The search criteria used on the GMrepo website included ‘phenotype’ (colitis and ulcerative) and ‘experiment type’ (Amplicon). The search keywords employed in EMBL-EBI and Google Scholar were “UC”, “16S”, “gut”, and “ulcerative colitis”. In Google Scholar, “bioproject” was added as the search keyword.

### Inclusion criteria

We included all studies involving gut microbiome composition in patients with confirmed UC, including controls without UC, which provided raw data on the 16S rRNA gene amplicon. We excluded projects involving animals or infants as experimental subjects, which had sampling sites on mucous membranes and did not provide detailed grouping information – the included studies comprised cohort, case-control, and cross-sectional studies. Finally, we incorporated datasets from 11 studies with the following National Center for Biotechnology Information (NCBI) Bioproject IDs: PRJEB33711, PRJNA50637, PRJDB6133, PRJNA368966, PRJNA431126, PRJNA596546, PRJNA681685, PRJNA753210, PRJNA541040 and PRJNA398089. Each study was conducted after receiving approval from the Institutional Review Board (IRB) at their institutes. In the data collection, because PRJNA50637 contained a large amount of UC data (*n* = 601) from the USA without HC, we added HC from PRJNA296920 and PRJNA386260 conducted in the USA.^19^ They were appropriate to use as HC for the present study since PRJNA296920 conducted inflammatory bowel disease research. The HC did not have any (inflammatory bowel disease, including UC. PRJNA386260 was a cross-sectional data set in the USA and was especially collected from healthy people for use in conjunction with other microbiome studies, so the HC did not include any IBD and UC.^18^ We also considered age when supplementing the HC data. The age groups with the highest incidence of UC are 20–30 and 50–80.^1^ The age of the healthy control subjects of PRJNA386260 was 55.3 ± 1.39, which coincided with the second-highest incidence time of UC. The data in PRJNA50637 comes from patients in the USA, and the use of PRJNA296920 and PRJNA386260 to supplement HC data also considers that both projects are derived from patient data in the USA.

### Metagenomic data analysis and downstream analysis

**Figure 6.**
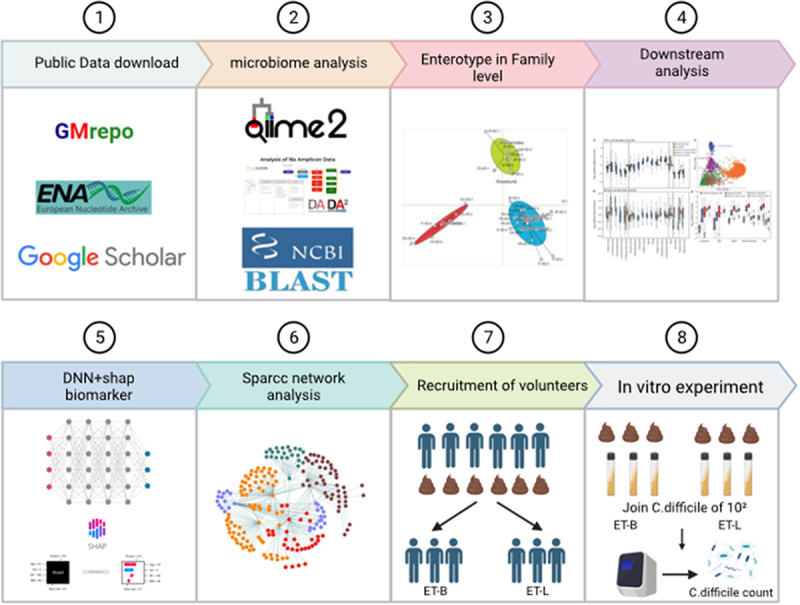
Workflow. Enterotype classification adopted by Arumugam et al.^9^ was used. ET-B: enterotype bacteridaceae, ET-L: enterotype Lachnospiraceae, ET-C: enterotype Clostridiaceae. Deep neural network (DNN). SHapley additive exPlanations (shap).

The selected data provided fecal fastq files, and some projects provided the age and gender of the participants. However, no projects included other potential confounders for UC risk, such as lifestyles. The fasta files analyzed were analyzed using the quantitative insights into microbial ecology (QIIME2, Accessed on February 2021) software package for 16S rRNA gut microbiome analysis.^36^ First, we used the Demux plugin to read and decomplex the paired-end sequences. Subsequently, the DADA2 pipeline was employed for sequence quality control and feature table construction, resulting in a unique sequence file.^37^ During the noise reduction filtering process in DADA2, sequences with forward and reverse median quality scores below 30 and sequence lengths shorter than 126 were filtered out. The 16S ribosomal RNA sequence database (https://ftp.ncbi.nlm.nih.gov/blast/db/) of NCBI’s basic local alignment search tool (BLAST)+ was used for sequence alignment to assign classifications to the feature data (unique sequences). After denoising, a classification was assigned to the representative sequences (Figure 6). While RDP, SILVA, and Greengenes are popular 16S rRNA databases, they often lack species-level resolution. Given that NCBI contains the most sequences and aligns best with SILVA’s taxonomic units, we chose it for our classification tasks.^38,39^

The operational taxonomic unit (OTU) table and the taxonomy file obtained through DADA2 and NCBI BLAST+ were merged into a single taxonomy for each classification. In further analysis, the counts of each OTU were used as they were, or in relative abundance, to normalize their counts according to the specified analysis. The relative abundance table for each taxonomic level was calculated. The q2-picrust2 plugin of QIIME2 was used to generate the Kyoto Encyclopedia of Genes and Genomes (KEGG) Ortholog (KO) abundance table, which was predicted by the functional orthologs of the gut microbiome. We then converted the KO abundance table into a relative abundance table and obtained the corresponding KO metabolic pathway maps through online comparison at https://www.genome.jp/kegg/. The KOs of related functions were combined and summed to determine the relative activity of various gut microbiome functions for each subject (Figure 6).

We employed the enterotype clustering method provided by Arumugam et al. in the published paper on enterotypes of the human gut microbiome and stratified the gut microbiome clustering according to the genus.^9^ To reduce noise in enterotype analysis, we used the *VAR* function in Excel to calculate the variance of each OTU in the genus relative abundance table and eliminated OTUs with a variance of less than 1. Then, we performed enterotype clustering using the R code provided at https://enterotype.embl.de/enterotypes.html. The optimal number of clusters was determined using the Calinski-Harabasz (CH) method. This step is also included in the intestinal clustering code provided by Arumugam et al.,^9^ specifically using the *index.G1* function in the clusterSim package. The Chi-square test was used to analyze the significance of the difference in the number of each enterotype. We calculated α-diversity (Shannon) using the relative abundance table classification at the level of species and β-diversity (Bray-Curtis) metrics using the R packages *vegan* and *ade4*. α-diversity (Shannon) was statistically analyzed using the Wilcoxon test. The Adonis function from the *vegan* package was used to determine significance based on the Bray-Curtis distance. The same procedure was applied to the total participants and those in ET-B and ET-L (Figure 6).

### DNN and SHAP

We divided the relative abundance data at the species level by the total participants and those in ET-B and ET-L. The DNN classification model was built using the Keras package (2.8.0) in Python. To reduce the noise caused by low-abundance species, we calculated and excluded the low-abundance OTUs with variance less than 0.1 in the OTU table, which may represent rare species or those with little impact on the study. We utilized the *StandardScaler* function from the scikit-learn package to perform z-score normalization on the data. Subsequently, using the *train_test_split* function from scikit-learn, we randomly divided the data into a training set (80%) and a test set (20%). Then, the *ttest_ind* function from the *scipy* package in Python was used for statistical testing of each OTU in the training set. Only OTUs significantly different between UC and HC were retained for DNN model training. Subsequently, we employed SHAP to compute the importance of each feature, retaining the top 20 significant taxons. We used the *ggpubr* package in R to conduct a Wilcoxon rank test on the 20 taxons and subsequently plotted box plots. We then retrained the model with this refined dataset. After training the model, its performance was evaluated on the test set using metrics such as the area under AUC, accuracy, sensitivity, specificity, precision, and the F1 score. The test data was randomly divided 1,000 times using repeated data splitting, and the mean and standard deviation of the test performance parameters were calculated. Concurrently, the receiver operating characteristic curve (ROC) of the model on the test set was plotted using the Matplotlib package in Python.

SHAP is a method for explaining the black-box characteristics of machine learning models.^40^ We used SHAP (0.39.0) to calculate the SHAP values of each feature for the DNN and determined the importance of the features and their impact on classification based on these values. We also performed a t-test for significance analysis. Since the ET-C cohort only included UC patients, constructing a DNN classifier was not possible. Therefore, the analysis was conducted for the total, ET-B, and ET-L cohorts (Figure 6). We noticed taxa with unclear categories in the stacked bar chart from the SHAP explainer. It could be due to uncertainties and noise when building the model. Therefore, we compared the average abundance of these taxa in UC and HC to determine their classifications.

### Network analysis and prediction of gut microbiome function

First, we used an OTU table for species that had not been converted to relative abundance and manually transformed it into the mothur (1.48.0) count_table format. Subsequently, the *make.shared* function was applied to adapt the data for downstream analysis in mothur.^41^ We then employed the *sub.sample* function, setting the size to 1000 and the persample to True. This step allowed us to normalize the data and, at the same time, exclude samples with a total count below 1000. The *sparcc* function was then used with all parameters set to their defaults to carry out the Sparse Correlations for Compositional Data (SparCC) analysis. Following this, we manually extracted the correlation matrix for the top 20 taxa identified from DNN and SHAP analysis. The resulting Sparse Correlation Network (SCN) was visualized using the Cytoscape 3.4.0 application. Edges without significant correlations or correlation coefficients below 0.1 were not displayed in the SCN.

Additionally, using the relative functional profile from PICRUSt2, we employed the native LEfSe tool available on GitHub (https://github.com/SegataLab/lefse) to calculate the LDA values between UC and HC for the metabolic functions that PICRUSt2 had predicted.^42^ Furthermore, we utilized the relative abundance tables for gut bacteria and metabolic functions from PICRUSt2 to compute their Pearson correlation coefficients. A correlation heatmap was generated using the *pheatmap* R package (version 1.0.12).

### Recruitment of experimental subjects and processing of fecal samples

Because UC patients have an increased risk of *Clostridium difficile* infection (CDI), CDI is the leading cause of UC disease progression, and the prevalence of UC continues to increase.^43^ Consistent with the theme of our study, in order to observe the resistance of different intestinal types to *C. difficile*, we designed and carried out a co-culture experiment. Healthy volunteers aged 20–30 years, regardless of gender, were recruited for this study according to the following conditions: No acute or chronic diseases, no antibiotic treatment, and no probiotic supplementation within 3 months prior to stool collection. Alcohol avoidance was advised the day before stool collection. The IRB at Hoseo University, Korea, reviewed and approved the study protocol, with the approval number 1041231-190816-BR-094-02. Written informed consent was obtained from all participants before initiating the study.

Upon arrival at the laboratory in the first round, each participant was given a centrifuge tube containing 10 mL of sterile water and a sterile swab. Participants were instructed to self-collect fecal samples in a private, sterile environment, such as a dedicated restroom facility provided within the laboratory premises. After collection, participants were to immediately place the sample in the provided centrifuge tube and return it to the laboratory. DNA extraction from the received samples was promptly conducted to ensure sample integrity and prevent possible contamination. Total DNA was extracted from fecal microbiomes using the QIAamp PowerFecal DNA Kit (QIAGEN, Hilden, Germany), and the concentration of the DNA samples was adjusted to 5 ng/μL using diethyl pyrocarbonate (DEPC, AM9906 Thermo Fisher, Waltham, MA, USA) water. Subsequently, V3-V4 gene fragments were amplified using the KAPA HiFi HotStart ReadyMix PCR Kit (KK2602; KAPA Biosystems, Wilmington, MA, USA) and primers B341F (5’-CCTACGGGNGGCWGCAG-3’) and B805R (5’-GACTACHVGGGTATCTAATCC-3’). The 16S DNA amplicons were then purified using AMPure beads (Beckman Coulter, Brea, CA, USA). Finally, the purified 16S RNA samples were sent to Macrogen Ltd (Seoul, Korea) for high-throughput sequencing.

We manually classified the enterotypes using the participants’ relative abundance table at the family level.^44^ We set a benchmark for manual partitioning of enterotypes. Subjects with a relative abundance of Bacteroidaceae greater than 35% were classified into the ET-B group. Those with a combined relative abundance of Lachnospiraceae and Ruminococcaceae greater than 25% were categorized into the ET-L group. According to the above criteria, subjects assigned to the ET-B and ET-L groups must not meet the conditions for the other group. In the second round of fecal sample collection, participants followed the same steps as in the first round but with a few key differences. Each participant was provided with an empty 50 ml centrifuge tube for fecal sample collection at this stage. Upon collection, the samples were immediately submitted to the laboratory. We accurately weighed 5 g of the fecal sample and introduced it into 25 ml of pre-prepared modified Brain Heart Infusion (mBHI) medium. The mixture was stirred and kept at 4°C for 1 minute. The supernatant (10 mL) was then collected by centrifuging the mixture at 225 ×g for 3 minutes at 4°C. The supernatant was used as a bacterial extraction solution and stored in a laminar flow hood. The collection time of the second round of fecal samples was controlled to be within 15 minutes of adding the medium.

### Culture of bacterial strains and co-culture

The commercial strain of *C. difficile* KCTC 5009 was purchased from the Korean Collection for Type Cultures (KCTC, 5009). The strain was anaerobically incubated at 37°C for 18 hours in an mBHI medium. The basal BHI culture medium (KisanBio, MB-B1007) was supplemented with 0.5% yeast extract (Sigma-Aldrich, Y1625) and 5% L-cysteine (Sigma-Aldrich, St. Louise, MO, USA). The culture was grown in Hungate anaerobic culture tubes. Nitrogen gas was introduced to displace the oxygen in the liquid, and then the tubes were moved into an anaerobic chamber. The culture medium was added to each tube, which was then capped and sterilized using an autoclave at 121°C for 15 minutes. The co-culture of *C. difficile* with fecal bacterial communities was also performed using the mBHI medium. Previous studies have shown that the mBHI medium for co-culturing fecal bacterial communities *in vitro* allowed the preservation of more microbial diversity compared to other media.^45^

After overnight culture, the *C. difficile* strain was counted using a bacterial counting plate (Marienfeld Superior, Lauda-Königshofen, Germany). We used fecal bacterial cultures from each individual as controls (ETL-Con, ETB-Con) and co-cultured with C. difficile 1 × 10^3^ as co-cultivation groups (ETL-CD, ETB-CD). The process for each sample was repeated three times. The cultures were incubated at 37°C, and samples were collected from the same tube at 12 and 24 hours for quantitative polymerase chain reaction (qPCR) quantification of the bacterial numbers.

### Primer design and qPCR Quantification

The whole-genome sequence of *C. difficile* KCTC 5009 was downloaded from the website https://kctc.kribb.re.kr. Primers were designed using the Species Primer automated high-throughput screening design-specific primer pipeline.^46^ The ntpG-1 gene expressed into V-type sodium ATPase subunit G was ultimately chosen as the qPCR target for *C. difficile*. The final primers designed were forward ATCTTCCCAGCCTATGATAGTGAC, and reverse TCGCATATTCATCTTCATCAACCAACT. After confirming the primer design, DNA was extracted from the overnight culture of *C. difficile* following the same protocol as fecal DNA extraction. The extracted DNA was diluted 10-fold with DEPC water for qPCR detection. Two replicates were used for each sample, using DNA, SYBR green, and forward and reverse primers.^47^ The qPCR quantification was performed using a PCR machine (Step One Plus; Applied Biosystem, Waltham, MA, USA), and gene expression levels in unknown samples were quantified using the comparative CT (ΔΔCT) method. The CT values obtained at different concentrations were used to construct a standard curve for quantifying the *C. difficile* colony-forming units. DNA was extracted from the samples collected during the culture process using the same method used for qPCR quantification.

### Statistical analysis

The ttest_ind function of Python’s scipy package was used for the t-test test between the two groups. Statistical analysis among multiple groups was performed using SPSS (20.0) for ANOVA (Tukey’s post hoc) analysis. The data are presented as mean ± standard deviation (SD), and statistical significance was set at *P* < 0.05.

## Supplementary Material

Supplementary Tables and figures.docx

## Data Availability

The datasets generated, and the script during the current study are available in the Github repository: https://github.com/WXG920713/Gut-microbes.
