# Supplementary material for: The impact of gut microbiome enterotypes on ulcerative colitis: identifying key bacterial species and revealing species co-occurrence networks using machine learning
Source: Gut Microbes. 2023 Dec 20;16(1):2292254. doi: 10.1080/19490976.2023.2292254 (PMC10761161; doi:10.1080/19490976.2023.2292254)

Supplementary Table S1. Deep Neural Network classification model performance measurements for classification of UC and HC participants based on different enterotypes

|  | AUC | Accuracy | Sensitivity | Specificity | Precision | F1 |
| --- | --- | --- | --- | --- | --- | --- |
| Total(n=1382) | 0.96±0.001 | 0.9±0.001 | 0.92±0.001 | 0.89±0.001 | 0.87±0.001 | 0.9±0.001 |
| ET-B(n=424) | 0.95±0.001 | 0.88±0.001 | 0.94±0.001 | 0.71±0.001 | 0.89±0.001 | 0.91±0.001 |
| ET-L(n=742) | 0.93±0.001 | 0.93±0.001 | 0.78±0.001 | 0.97±0.001 | 0.86±0.001 | 0.81±0.001 |
| Values were randomly sampled from the test set for 1000 iterations and mean±SD was calculated. AUC: Area Under Curve, UC: Ulcerative colitis, HC: Health control, Total: Model trained using overall data, ET-B: Model trained using data labeled Bacteroidaceae enterotype, ET-L: Model  trained using data labeled Lachnospiraceae enterotype. | | | | | | |

Supplementary Table S2. The sum of positive and negative connections and correlation coefficients of each bacteria in total Species Co-occurrence Network

| Name | Positive-cor^a^ | Negtive-cor^b^ | Correlation^c^ |
| --- | --- | --- | --- |
| *Alistipes putredinis* | 17 | 14 | 5.686089 |
| *Anaerostipes hadrus* | 18 | 15 | 5.85539 |
| *Bacteroides uniformis* | 17 | 15 | 5.867939 |
| *Blautia faecis* | 18 | 15 | 6.373361 |
| *Blautia luti* | 18 | 15 | 5.088966 |
| *Collinsella aerofaciens* | 16 | 14 | 3.90642 |
| *Erysipelatoclostridium ramosum* | 17 | 1 | 4.623417 |
| *Faecalibacterium prausnitzii* | 17 | 15 | 6.852711 |
| *Fusicatenibacter saccharivorans* | 18 | 15 | 6.474161 |
| *Gemmiger formicilis* | 18 | 15 | 6.2736 |
| *Lachnospira eligens* | 18 | 15 | 6.010284 |
| *Loriellopsis cavernicola* | 11 | 0 | 1.433097 |
| *Oscillibacter valericigenes* | 18 | 15 | 4.989794 |
| *Parabacteroides merdae* | 18 | 15 | 4.961917 |
| *Phocaeicola vulgatus* | 16 | 14 | 3.908034 |
| *Eubacterium rectale* | 16 | 13 | 4.348852 |
| *Ruminococcus gnavus* | 17 | 2 | 3.947398 |
| *Roseburia intestinalis* | 12 | 10 | 2.354696 |
| *Akkermansia muciniphila* | 14 | 12 | 3.490726 |
| In order to quantify the connection relationship of each bacteria, we calculated the number of connections of each bacterium in the analysis and only included connections with a Sparcc correlation coefficient greater than 0.1 in the calculation. a. The number of positively related connections of each bacteria b. The number of negatively related connections of each bacteria c. The value was obtained by adding the absolute values of the correlation coefficients greater than  0.1 associated with each bacterium. | | | |

Supplementary Table S3. The sum of positive and negative connections and correlation coefficients of each bacteria in ET-L Species Co-occurrence Network.

| name | Positive-cor^a^ | Negtive-cor^b^ | Correlation^c^ |
| --- | --- | --- | --- |
| *Anaerobutyricum hallii* | 12 | 12 | 2.574434 |
| *Bacteroides uniformis* | 13 | 10 | 2.404105 |
| *Lachnoclostridium pacaense* | 11 | 3 | 1.886864 |
| *Lawsonibacter asaccharolyticus* | 11 | 7 | 2.62524 |
| *Monoglobus pectinilyticus* | 9 | 6 | 1.593985 |
| *Neglectibacter timonensis* | 12 | 8 | 2.292604 |
| *Odoribacter splanchnicus* | 13 | 9 | 2.557858 |
| *Papillibacter cinnamivorans* | 11 | 8 | 2.200008 |
| *Romboutsia timonensis* | 10 | 8 | 1.991382 |
| *Clostridium leptum* | 5 | 4 | 0.651962 |
| *Clostridium spiroforme* | 8 | 2 | 1.541442 |
| *Anaerostipes hadrus* | 9 | 8 | 1.875086 |
| *Bifidobacterium adolescentis* | 5 | 4 | 0.809678 |
| *Blautia obeum* | 12 | 10 | 2.787276 |
| *Coprococcus comes* | 9 | 6 | 1.702366 |
| *Dorea formicigenerans* | 14 | 7 | 2.726435 |
| *Lachnospira eligens* | 8 | 7 | 1.628422 |
| *Prevotella copri* | 5 | 2 | 0.694209 |
| *Akkermansia muciniphila* | 11 | 9 | 1.865224 |
| In order to quantify the connection relationship of each bacteria, we calculated the number of connections of each bacterium in the analysis and only included connections with a Sparcc correlation coefficient greater than 0.1 in the calculation. a. The number of positively related connections of each bacteria b. The number of negatively related connections of each bacteria c. The value was obtained by adding the absolute values  of the correlation coefficients greater than 0.1 associated with each bacterium. | | | |

Supplementary Table S4. The sum of positive and negative connections and correlation coefficients of each bacterium in ET-B Species Co-occurrence Network.

| name | Positive-cor^a^ | Negtive-cor^b^ | Correlation^c^ |
| --- | --- | --- | --- |
| *Bacteroides fragilis* | 12 | 6 | 1.861545 |
| *Bacteroides uniformis* | 18 | 13 | 3.935834 |
| *Blautia faecis* | 16 | 12 | 3.522779 |
| *Blautia luti* | 13 | 10 | 2.369025 |
| *Enterocloster clostridioformis* | 14 | 4 | 3.506803 |
| *Fusicatenibacter saccharivorans* | 15 | 12 | 3.45416 |
| *Gemmiger formicilis* | 13 | 11 | 2.634131 |
| *Monoglobus pectinilyticus* | 14 | 11 | 2.74156 |
| *Neglectibacter timonensis* | 5 | 4 | 0.675438 |
| *Odoribacter splanchnicus* | 14 | 12 | 2.11092 |
| *Oleidesulfovibrio alaskensis* | 9 | 7 | 1.354265 |
| *Oscillibacter valericigenes* | 9 | 8 | 1.527654 |
| *Ruminococcus gnavus* | 10 | 2 | 1.937546 |
| *Bifidobacterium longum* | 6 | 6 | 0.94542 |
| *Lachnoclostridium pacaense* | 7 | 7 | 0.89515 |
| *Desulfovibrio simplex* | 8 | 4 | 1.516559 |
| *Hydrogenophilus thermoluteolus* | 3 | 2 | 0.330605 |
| *Phocaeicola dorei* | 5 | 4 | 0.978458 |
| *Alistipes shahii* | 13 | 10 | 2.386936 |
| *Anaerostipes hadrus* | 10 | 9 | 1.992298 |
| In order to quantify the connection relationship of each bacteria, we calculated the number of connections of each bacterium in the analysis and only included connections with a Sparcc correlation coefficient greater than 0.1 in the calculation. a. The number of positively related connections of each bacteria b. The number of negatively related connections of each bacteria c. The value was obtained by adding the absolute values of  the correlation coefficients greater than 0.1 associated with each bacterium. | | | |


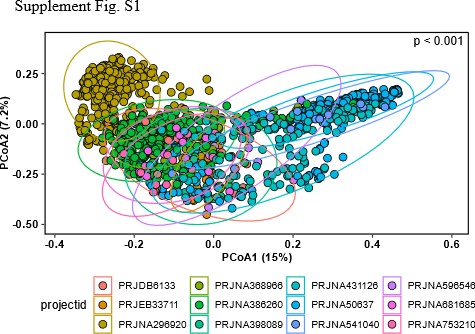


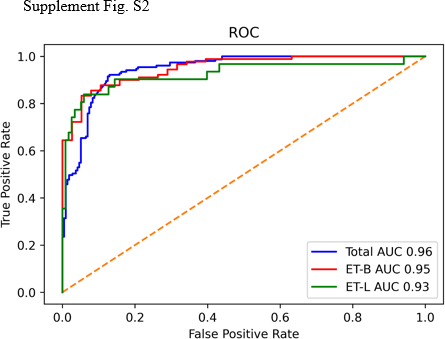


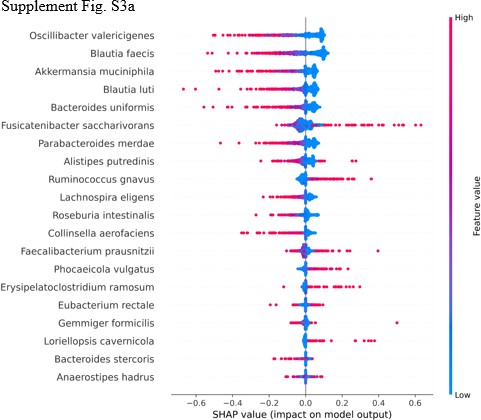


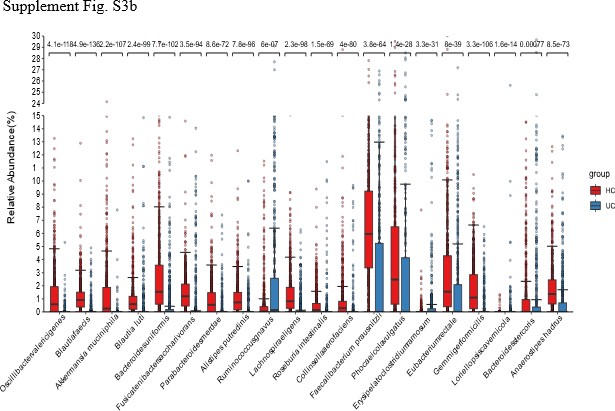


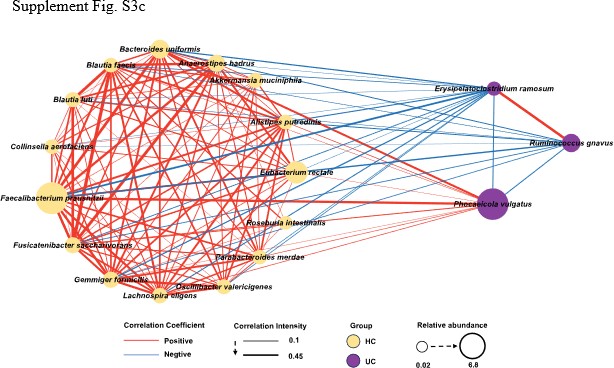


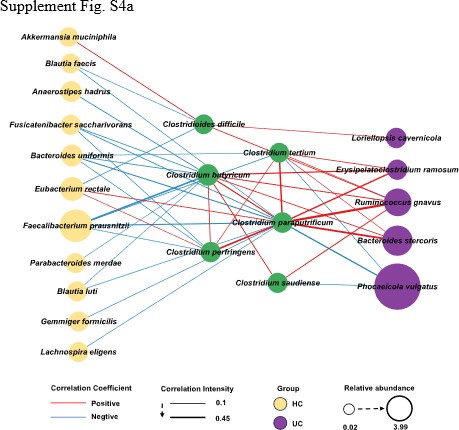


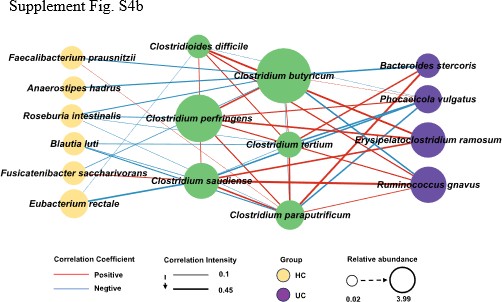

Supplement: Supplementary Tables and figures.docx [file KGMI_A_2292254_SM7321.docx]
